# A review of extensive variation in the design of pitfall traps and a proposal for a standard pitfall trap design for monitoring ground‐active arthropod biodiversity

**DOI:** 10.1002/ece3.2176

**Published:** 2016-05-12

**Authors:** Grant R. Brown, Iain M. Matthews

**Affiliations:** ^1^ Centre for Biological DiversityUniversity of St AndrewsSir Harold Mitchell BuildingSt AndrewsKY16 9THUK

**Keywords:** Araneae, biodiversity sampling, carabidae, formicidae, pitfall trap, standard design

## Abstract

To understand change in global biodiversity patterns requires large‐scale, long‐term monitoring. The ability to draw meaningful comparison across studies is severely hampered by extensive variation in the design of the sampling equipment and how it is used. Here, we present a meta‐analysis and description highlighting this variation in a common, widely used entomological survey technique. We report a decline in the completeness of methodological reporting over a 20‐year period, while there has been no clear reduction in the methodological variation between researchers using pitfall traps for arthropod sampling. There is a growing need for improved comparability between studies to facilitate the generation of large‐scale, long‐term biodiversity datasets. However, our results show that, counterproductive to this goal, over the last 20 years there has little progress in reducing the methodological variation. We propose a standardized pitfall trap design for the study of ground‐active arthropods. In addition, we provide a table to promote a more standardized reporting of the key methodological variables. Widespread adoption of more standardized methods and reporting would facilitate more nuanced analysis of biodiversity change.

## Introduction

Ongoing loss of biodiversity is a global issue, necessitating investigation at multiple spatial and temporal scales (Magurran et al. 2010; Keil et al. 2012; Dornelas et al. 2014; Stein et al. 2014). “Big data” generated from multiple researchers’ efforts is likely to become ever more important in unveiling the scope of biodiversity change. This is especially relevant when this change concerns taxonomically difficult organisms (Peters et al. 2014). The importance of long‐term, standardized data collections has been highlighted in several recent publications (Fischer et al. 2010; Magurran et al. 2010; Dornelas et al. 2014). However, there exist significant difficulties in the analysis of long‐term and spatially large data, especially where the methodology between researchers differs (Gotelli and Colwell 2001, 2011). Lack of comparability across studies has recently been highlighted in other fields (Alivisatos et al. 2015) and is likely to become an emerging issue more widely.

One solution to the difficulty of comparing between smaller research projects is to rely on statistical methods to control for between‐researcher idiosyncrasies, and approaches such as rarefaction have been used to allow comparison of species richness when sampling effort differs in this manner (Engemann et al. 2015). However, this approach is not without its own share of potential pitfalls (Gotelli and Colwell 2001). A second option is to adopt standardized methods for data collection. This has been more rarely achieved, but there do exist collaborative studies where the use of identical methodology has been used in order to tackle research questions at larger spatial scales (Niemelä et al. 2002). In other cases, industrial standards are used to ensure large‐scale standardized methodology (e.g., Levan 2015). One established example is River Invertebrate Prediction and Classification System (RIVPACS), which was designed specifically in response to the lack of comparability in the United Kingdom's National River Survey program during the 1970s (Centre for Ecology and Hydrology, 2015). RIVPACS allows comparison of freshwater invertebrate assemblages and assessment of river health and relies on a standardized methodology where even details such as the dimension of the nets used to kick sample is controlled.

A standardized methodology would deliver a number of benefits. For example, collaborative research across larger global or temporal scales than is achievable for a single researcher becomes more straightforward. Long‐term or large spatial scale analyses often have considerable “noise” in the first place, so any reduction in this background variation is going to increase the chances of detecting ecological signals and reduce the need for complex analytical approaches (Niemelä et al. 2002; Magurran et al. 2010; Dornelas et al. 2014). Further, a standard technique facilitates exploration of associated biases without the need to encompass a potentially infinite number of alternate designs. As use of a standardized methodology increases, the ability to then compare against the “experimental norm” will also increase, making unusual or aberrant results easier to detect. The reporting of methodology can also be streamlined and the repeatability and analysis of the research simplified as a body of robust approaches is developed. Training in the technique can also be accelerated without the need for new researchers to invest considerable time in exploring the subtleties of the technique in order to become proficient.

**Figure 1:**
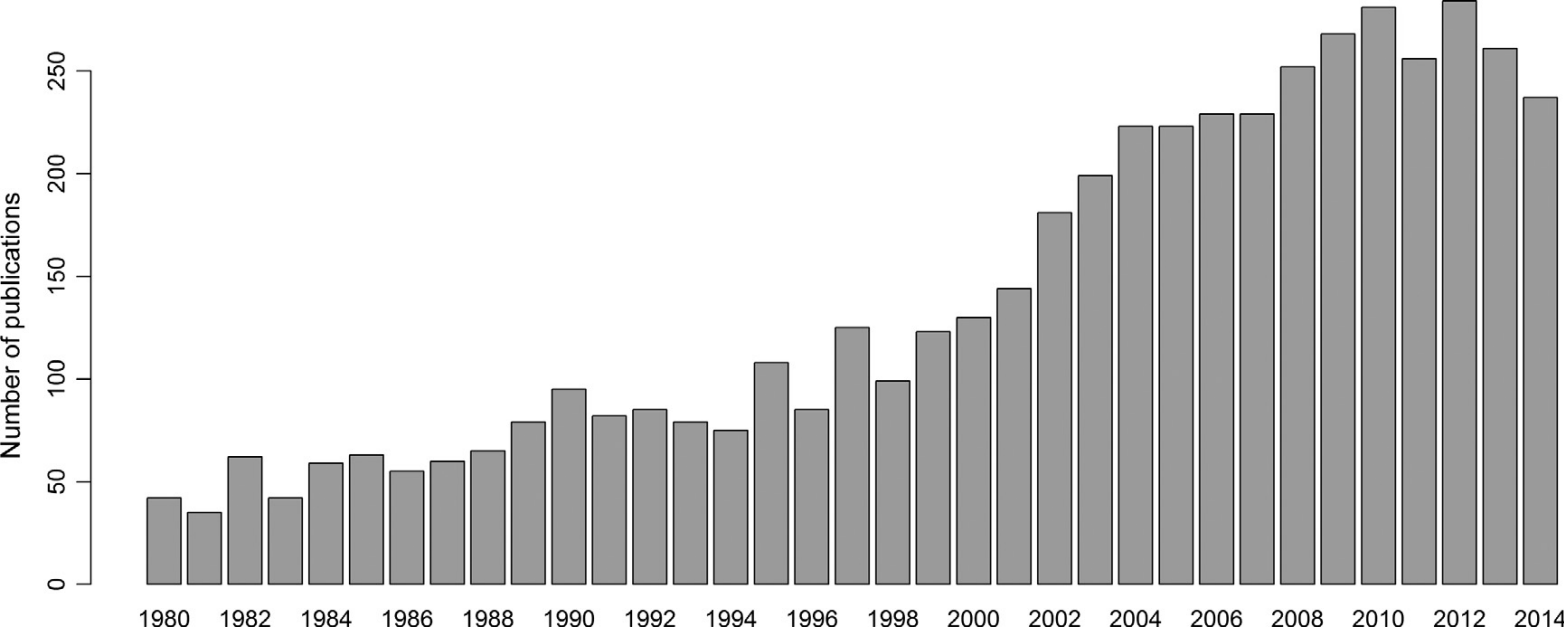
Number of hits per year for the search term “pitfall trap arthropod” using Thomson Reuters Web of Knowledge, between the years 1980 and 2014 (with lemmatization=on). Search conducted 30th November 2015. A total of 13,027 hits were returned from this year range.

In this review, we propose a standardization to a commonly used entomological apparatus; the pitfall trap. Pitfall trapping is often the sole method used to characterize ground‐active arthropod assemblages (e.g., Buddle et al. 2006; Knapp et al. 2013), and the number of publications featuring pitfall trapping is on the rise (Fig. 1). We focus on the design of pitfall traps rather than the experimental theory (spatial arrangement, number of samples, etc.) for several reasons; firstly, as we show, the design of pitfall traps varies considerably between researchers yet many features of pitfall trap design have been shown to significantly influence the capture rates of different taxonomic groups, sexes, and life stages (Luff 1975; Schmidt et al. 2006; Yamashita et al. 2010). Secondly, while some authors have published on how sampling design influences interpretation when using pitfall traps (e.g., Ward et al. 2001; Perner and Schueler 2004; Baker and Barmuta 2006), the interactions between trap design and sampling arrangement are still unclear. We will describe variation in the sampling design (number of traps used, duration of sampling, etc.), but we will not recommend a standardized sampling design, as we feel that standardization of the physical parameters of trap design is a necessary prerequisite of the standardization of the wider method.

This review therefore has four aims. Firstly, to provide a meta‐analysis of recently published papers to highlight the extent of variation in the design of pitfall traps when used for arthropod sampling. Secondly, to review the previous literature focusing on design features of pitfall traps. Thirdly, we explain the rationale for our proposed standardized pitfall trap design. Finally, this article aimed to stimulate discussion of the variation in methodology reporting and make suggestions on which features should be reported to enhance future repeatability and comparison of the research.

## Pitfall Trapping for Collecting Ground‐Active Arthropods

Pitfall traps are a commonly used technique for sampling ground‐active arthropods, and several reviews have discussed the relative biases and potential interpretation issues that may be encountered (Southwood 1978; Adis 1979; Woodcock 2005); for brevity these will not be discussed here in detail. In basic terms, however, pitfall traps can be used to generate an estimate of “activity‐density” – that is, the abundance of each species as a reflection of its activity during the sampling period and the density of the population in the sampled habitat. Activity is influenced by various factors such as the weather (Saska et al. 2013), although even this will likely vary by taxa as pitfall‐like traps have still managed to collect various arthropods running around under snow (Steigen 1973). For researchers interested in obtaining reliable species densities, pitfall trapping can be problematic and other methods are likely more suitable (Topping and Sunderland 1992). However, given that comparison of biodiversity often focus on species richness and assemblage distribution patterns (Gotelli and Colwell 2001), the relation of a particular species to exact densities is likely of lower importance than simply generating a reasonably unbiased snapshot of the relative abundances of the assemblage. In this regard, the use of pitfall traps is a satisfactory method and often collects more species than other sampling methods (Churchill 1993; Churchill and Arthur 1999), even although it may not capture all the species of the ground‐active arthropod guild in the environment (Driscoll 2010).

## Variation in the Design of Pitfall Traps

Various designs of pitfall traps for collecting ground‐active arthropods have been experimented with as the first pitfall‐type traps were used by Dahl 120 years ago (Dahl 1896). While there has been considerable debate over the limitations and advantages of the technique (Luff 1975; Southwood 1978; Topping and Sunderland 1992; Saska et al. 2013), it remains a widely used method in ecological research (Fig. 1). Despite widespread adoption, or perhaps because of this, there exists extreme variation in how the technique is used, reported and in how captures are interpreted (Adis 1979; Topping and Sunderland 1992; Work et al. 2002). Several authors have mentioned the need for standardization in the use of pitfall traps (Adis 1979; Koivula et al. 2003; Hancock and Legg 2012; Radawiec and Aleksandrowicz 2013). However, in the absence of cohesive recommendations, there seems to be no obvious move toward standardization and, as shown in our meta‐analysis, many researchers continue to use a near unique assortment of trap design features. We suggest that lack of standardization, coupled with varying completeness of methodological reporting, are among the biggest weaknesses associated with the use of pitfall traps in biodiversity monitoring and assessment.

One possible reason for the lack of standardization in the design of pitfall traps is that a clear “optimal design” has yet to be proposed. Recent reviews of the technique have tended to describe much of the existing variation in the technique without making firm recommendations, leaving those wishing to use pitfall traps to decide their own course of action (Woodcock 2005; Skvarla et al. 2014). There are occasions where valid reasons exist for allowing the design of traps to be tailored to a specialized research purpose. For example, Lehmitz et al. (2012) investigated the dispersal of Oribatid mites using small “mini‐pitfall” traps (of diameter 10 mm) and the adoption of a “standard biodiversity pitfall trap” of larger size would have likely negatively impacted their study aims, or at the very least resulted in considerable bycatch of nontarget organisms. However, the needs of specialized research should not constrain the ability of other researchers to standardize.

Biodiversity is a comparative discipline (Magurran 2004), and if one assumes the goal of most applied biodiversity investigation is to essentially generate representative measures of species richness and species abundance distributions (be it in different habitats, under different management regimes, in the face of climatic change, etc.), then the advantage of a standard design becomes more apparent in that such data can be more directly compared. The difficulties in quantifying species richness and biodiversity in general have already been discussed in other reviews (Gotelli and Colwell 2001; Magurran 2004), but broadly arise from issues relating to differences in sampling effort and duration, detectability of organisms and repeatability of the research – the exact issues that a standardized trap design would help begin to address.

## Quantifying the Existing Variation in the Design of Pitfall Traps

We used two approaches to highlight the variation in the pitfall trapping technique; a traditional review of the literature concerning specific aspects of pitfall trap design, and a meta‐analysis of 60 peer‐reviewed research papers that used pitfall trapping as either their main or only sampling method. The traditional review approach focused on pitfall trap methodology papers. We used this to inform our proposal for the standard pitfall trap, based on the findings of previous authors and likely future trends (e.g., the use of plastic rather than glass for pitfall trap construction is unlikely to be reversed).

To select papers for the meta‐analysis, we used the search term “pitfall trap arthropod” with results sorted “newest–oldest”. We selected a total of 60 peer‐reviewed papers published during 1994–2014. We selected 20 publications each from 2004 and 2014, 13 from 1994, and 7 published during 1995 (we could not access enough publications from 1994 and as such the papers from 1994 and 1995 were pooled into a single class, hereafter called “1994” for simplicity). The surveyed literature was published in 41 peer‐reviewed journals and conducted in 26 countries. The impact factor of the journals ranged from 0 (awaiting assessment) to 6.53, with a median of 1.55. Papers used in the meta‐analysis are listed in the supplementary material (Table S1).

Journals to which we did not have institutional access and conference proceedings were ignored. We collected information on the journal title and impact factor, where and why the study was conducted, the timing and duration of the research, as well as a number of other variables relating to the pitfall trap design (Table S2). Where papers referred to another paper for methodology, we noted this but scored the paper based on what was actually reported. Where a variable was not reported, we scored this as either “Not Stated” or “Not Applicable” (e.g., if a study aim was to collect live specimens, then the value for “preservative type” was “Not Applicable”).

**Figure 2:**
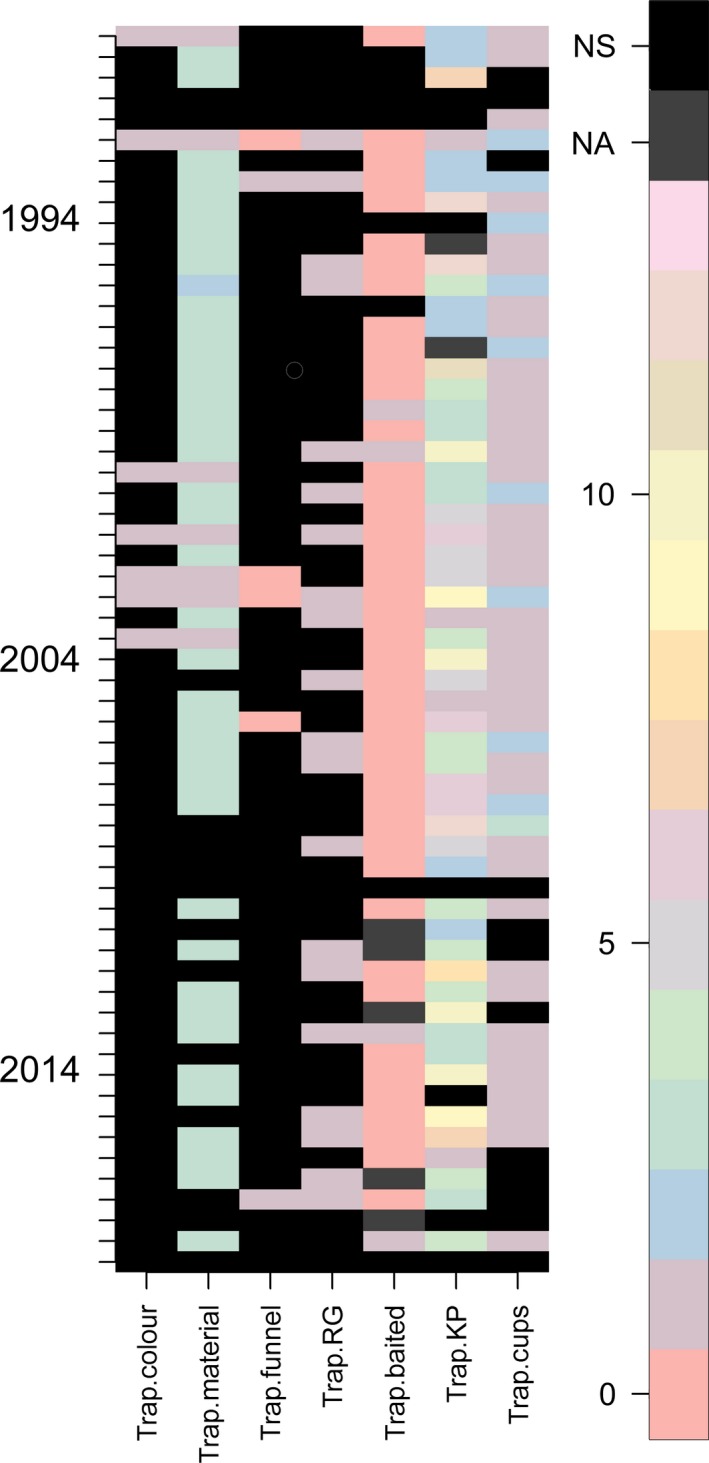
A colorized matrix of pitfall trap design variables recorded from 60 research papers. Different colors represent different categories of value (e.g., different types of killing preservative). Each row represents a single study, while each column details the particular variable as reported in that study. “0” scores indicate where a particular feature was not used by the authors, while details that were not reported were coded as category 15 (black) and “Not Applicable” as category 14 (dark gray). The color of pitfall traps and the use of a funnel were less frequently reported than the other variables throughout the 20‐year period. The number of papers not reporting design features increased from 1994 to 2014 (138 “NS”, 157 “NS”, respectively). The variables *trap.funnel*,* trap*.*RG*,* trap.baited,* and *trap.cups* were scored as either “were present = 1”, “were absent = 2,” or “changed during study = 3”. The variable *trap*.*KP* (killing preservative) was scored 0–12, corresponding to different categories of major additive (e.g., ethanol, propylene glycol, formalin), with an additional category (13) when the preservative was changed during the study. The variable *trap.material* was scored as follows: glass = 1, metal = 2, plastic = 3. The key interpretation point for these figures is that if methodological variation was zero (and reporting complete) between researchers, the figure would be of a uniform color.

**Figure 3:**
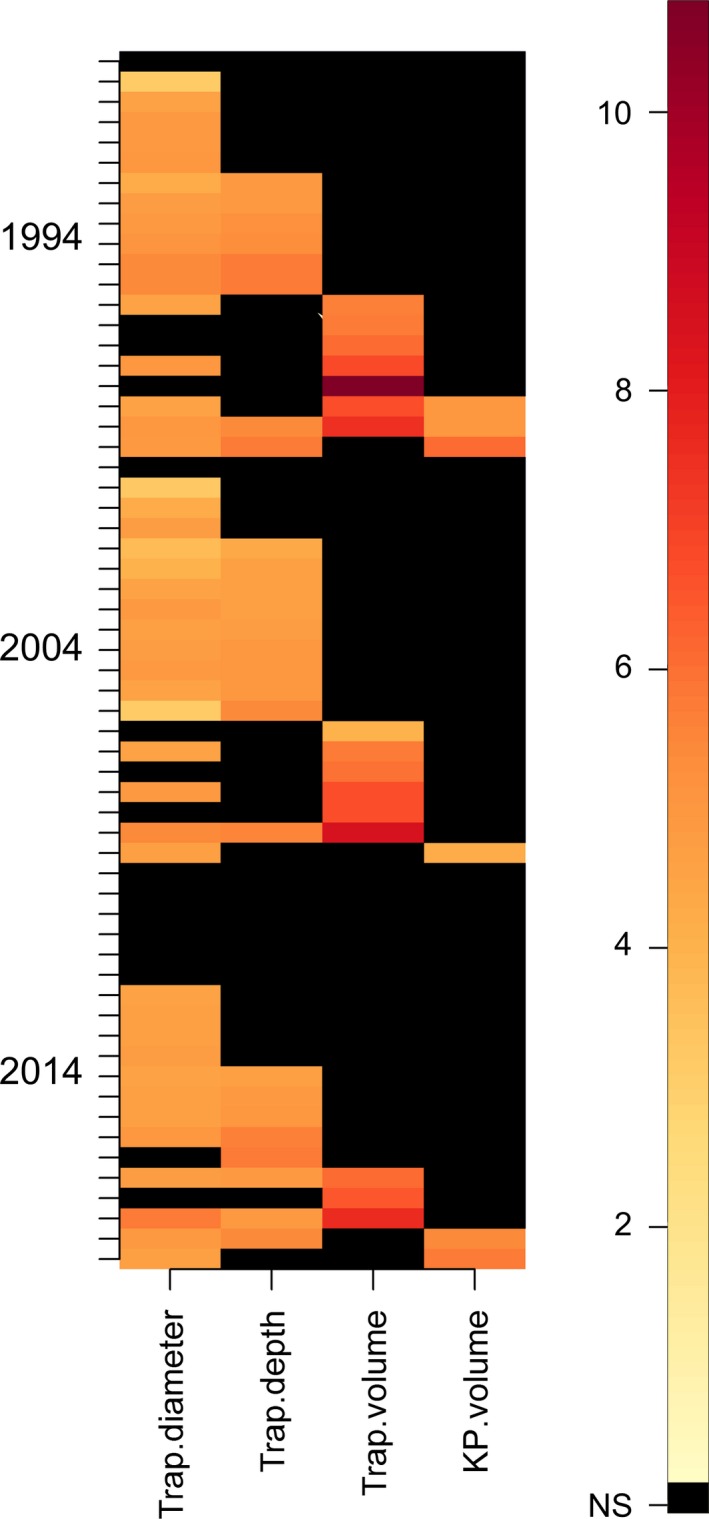
A colorized matrix of the pitfall trap design variables recorded from the 60 research papers. “Not stated” and “Not applicable” values are shown in black (“NS”). The darker colors represent higher values (e.g., larger diameter in mm). All values were transformed prior to plotting (log_e_ + 1). The volume of the trap and the volume of killing preservative used were less frequently reported than the diameter and depth of the pitfall trap. Several studies in the 2014 year class did not report the trap diameter, depth, volume, or killing‐preservative volume used. The key interpretation point for these figures is that if methodological variation was zero (and reporting complete) between researchers, the figure would be of a uniform color.

**Figure 4:**
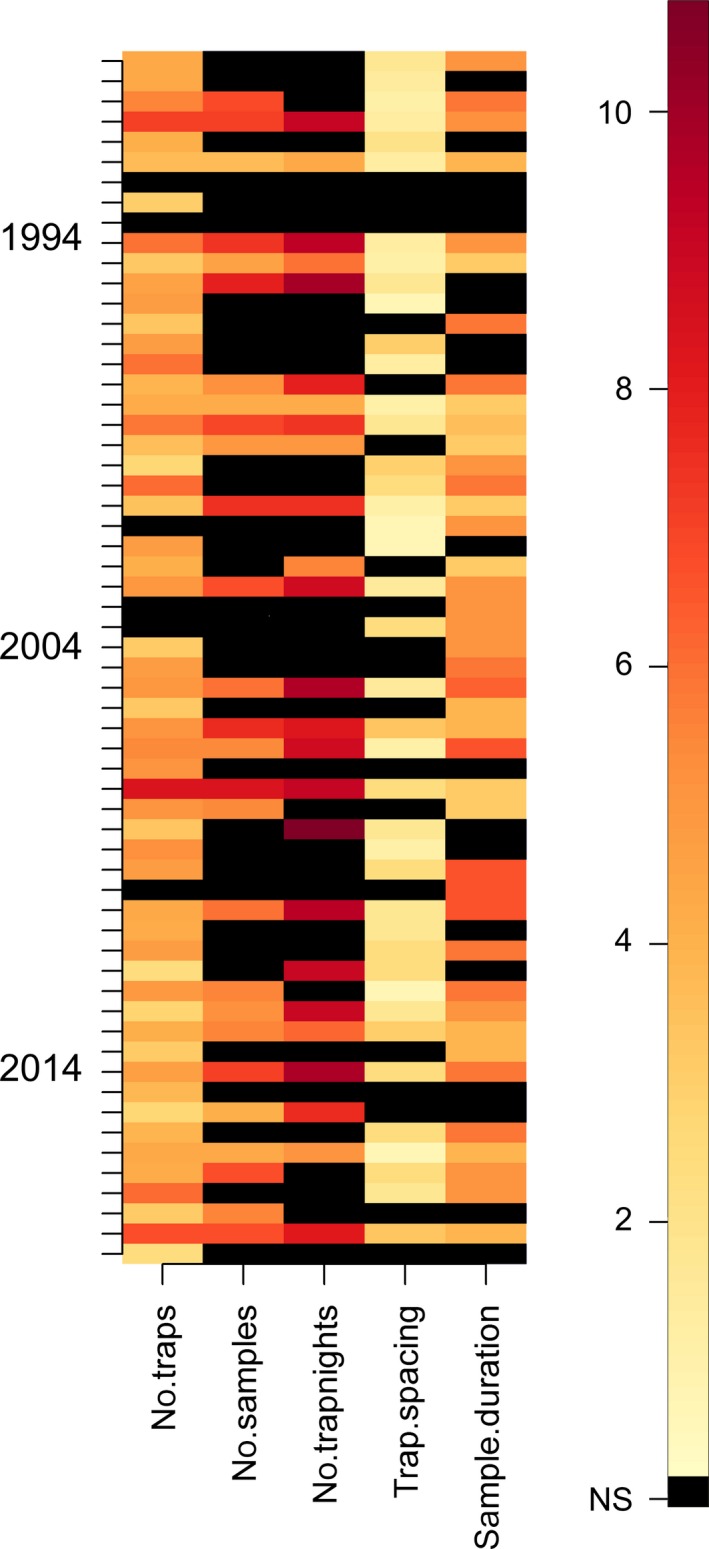
A colorized matrix of the pitfall trap sampling variables recorded from the 60 research papers. “Not stated” and “Not applicable” values are shown in black (“NS”). Darker colored bars represent higher values. All values were transformed prior to plotting (log_e_ + 1). While we make no recommendations regarding sampling design, it is worth noting that there was substantial variation in the number of traps used, the number of samples collected, and other factors such as the intertrap spacing and duration of individual sampling events. The key interpretation point for these figures is that if methodological variation was zero (and reporting complete) between researchers, the figure would be of a uniform color.

**Figure 5:**
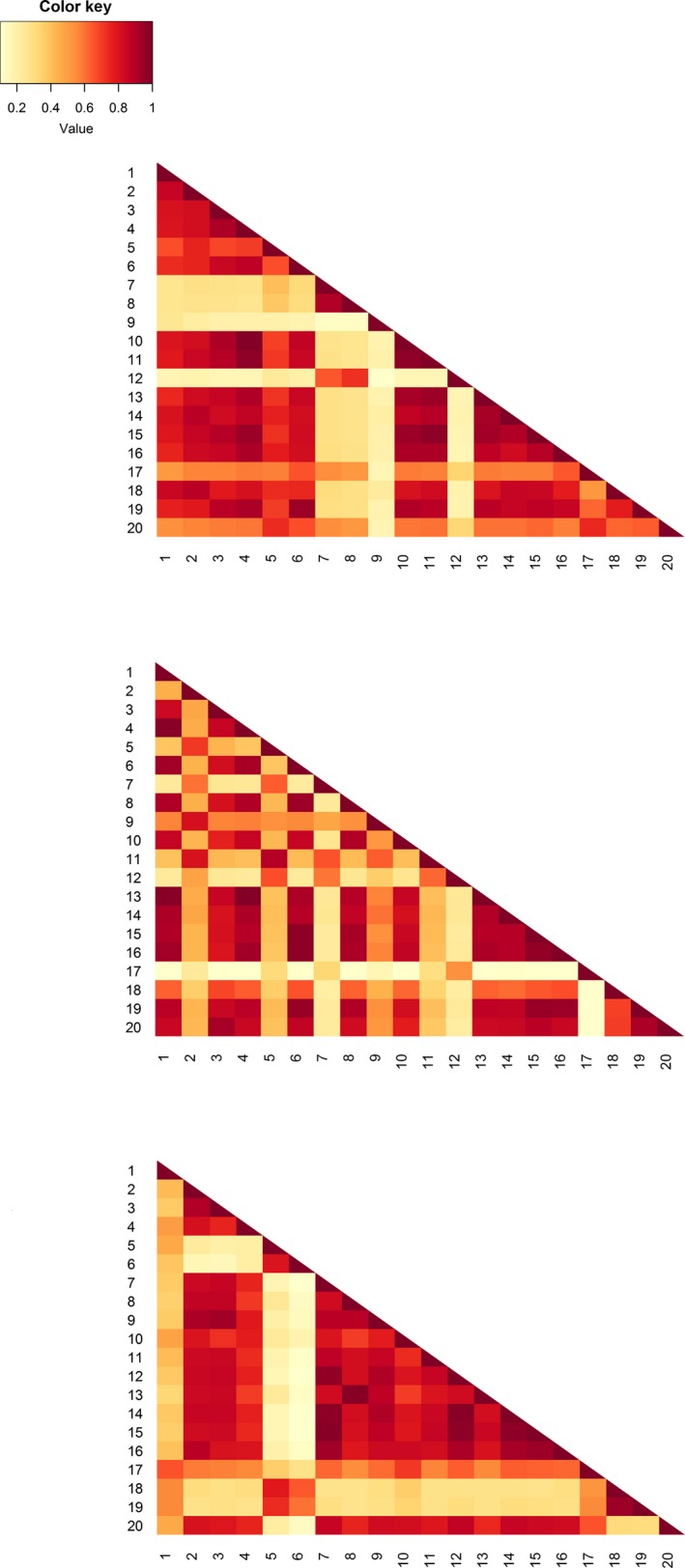
Colorized matrices of Bray–Curtis similarity between studies and between year groups based on their reported pitfall trap designs (from top to bottom: 1994, 2004, 2014). Each research paper was treated as a “site”, while the design variables (e.g., trap diameter, killing preservative used) were treated as “species”; the value of each variable (e.g., the trap diameter in mm) represented “abundance”. Each colored box is the similarity between two studies on the basis of their pitfall trap designs, with darker colors indicating more similarity. The apparent increase in similarity in the 2014 year group is likely due to the higher incidence of “NS” in the data. The key interpretation point for these figures is that if methodological variation was zero (and reporting complete) between researchers, the figure would be of a uniform color.

We used this information to produce figures showing the variation in a qualitative manner (Figs. 2, 3, 4) using the “myImagePlot” function in R (R Core Team, 2014) and based on a script by Chris Seidel (Seidel 2015). The details of how variables were scored are given in the supplementary material (Table S2). In order to obtain a more quantitative measure of the variation between studies based on the design of pitfall traps, we then used the function “betadiver” in the R package vegan (Oksanen et al. 2015) to generate a matrix of Bray–Curtis similarity scores based on pairwise comparisons between studies in each year class. We treated studies (rows) as “sites” and columns (pitfall trap design variables) as “species”, while the measure of each design feature represented “abundance” (e.g., the diameter of pitfall trap in mm). The matrices of beta‐diversity (variation) between studies were then colorized using the heatmap.2 function from the package gplots (Warnes et al. 2015; Fig. 5). The key interpretation point for these figures is that if methodological variation was zero (and reporting complete) between researchers, the figures would be of a uniform color.

The goal of this survey was not to critique experimental design or assess the validity or importance of the research, but simply to attempt to quantify the variation in the technique in terms of trap design and the rates of reporting of design features that have been shown to, or are likely to, influence capture rates of different arthropod taxa and therefore be expected to increase the difficulty of comparison between researchers.

## Trap Material

Pitfall traps have been constructed from a variety of materials, such as glass (Barber 1931), metal (Hertz 1927; Fichter 1941), and plastic (Fig. 2). The construction material has long been known to influence the rate of capture and subsequent retention of samples when used without a preservative (Luff 1975), although when a killing preservative is used this difference in capture rates reduces (Waage 1985). In the surveyed literature, the overall rate of reporting of pitfall trap material was 76%. From the studies that reported trap material, 82% used plastic to construct their traps. The highest incidence of nonreporting was found in the 2014 literature, with 45% of studies not reporting the material from which traps were constructed. In the context of standardization of future research, the use of plastic is preferable. Plastic containers are easily available and have been most commonly used in recent years. For field use, plastic is also lighter, less fragile, and cheaper to replace than glass traps. Additionally, the use of plastic throughout allows for more complex pitfall trap designs (e.g., use of funnels) that would be prohibitive to produce using glass.

## Trap Color

The behavior of various arthropod taxa to specific colors has previously been exploited by entomologists using sampling techniques such as pan trapping (Vrdoljak and Samways 2012). The influence of the color of the trap on captures has only recently been examined (Buchholz et al. 2009). The historical precedent for using glass likely eliminated the need for such consideration. As the visual acuity and color perception of many species differs (or is unknown), the effect of trap color is likely to be inconsistent between species and difficult to predict (Land 1997). Buchholz et al. (2009) found that, for spiders and Carabid beetles, white or yellow pitfall traps caught significantly different abundances compared to traps that were brown or green. Bees and flies were also caught at higher abundances in these brightly colored pitfall traps, possibly due to their similarity to floral coloration. Buchholz et al. (2009) suggested the use of white pitfall traps to increase efficiency for those interested in spiders and beetles and that bycatch could be analyzed by other researchers or taxonomists. However, we diverge from the recommendation of Buchholz et al. (2009) and recommend the use of transparent pitfall traps instead.

Work et al. (2002) discussed the hypothesis that variation in pitfall captures may be determined by differences in “background heterogeneity” (i.e., certain taxa may be captured at different rates due to the differential ability of these organisms to detect traps from background habitat, either by visual, tactile or chemosensory cues). Halsall and Wratten (1988) also suggested that differential ability to perceive edges of traps may account for some of the interspecies differences in trapping efficiency. As color may influence some taxa and not others, and this effect is itself inconsistent between and within taxa, it seems preferable to suggest the use of transparent pitfall traps to avoid introducing a known positive sampling bias active on only certain taxa. It is also worthwhile considering that the certain colors of pitfall traps (and rain guards in particular) are potentially attractive to small mammals and birds due to their contrast against the surrounding substrate (Schmidt et al. 2004). This may result in increased bycatch of nontarget vertebrate organisms or increased pitfall trap disturbance.

The recommendation of the use of transparent pitfall traps is further supported by the observation that bycatch is often not retained (pers. obs.), and there is a continuing decline in the number of willing, trained taxonomists able to process such material (Hopkins and Freckleton 2002). From the meta‐analysis, we found that only 11.6% of publications reported the color of pitfall traps used (Fig. 2).

## Use of Funnels

Several authors have utilized funnels with pitfall traps in an effort to increase capture efficiency, reduce vertebrate bycatch or reduce evaporation of killing preservatives (Fichter 1941; Obrist and Duelli 1996; Pearce et al. 2005; Lange et al. 2011; Radawiec and Aleksandrowicz 2013). However, analytical difficulties from comparison of different trap types (Gotelli and Colwell 2001) have until recently made quantitative comparison between funnel and nonfunnel pitfall traps difficult. Lange et al. (2011) reported that species richness between funnel and nonfunnel traps for beetles and spiders was not significantly different, but that funnel traps, and those of smaller diameter opening, caught significantly fewer small mammals. A similar reduction in vertebrate bycatch when comparing funnel and nonfunnel pitfall trap designs was reported by Radawiec and Aleksandrowicz (2013) concerning *Lacerta sp*. lizards. On the basis of reducing bycatch while apparently not adversely affecting the species richness of commonly sampled taxa, the use of funnels is supported and we propose that the standardized design for biodiversity sampling makes use of a funnel. Besides ethical arguments to reduce bycatch, the capture of small vertebrates is a problem for pitfall trapping in that they will either foul samples or alter the “attractiveness” of individual pitfall traps to some taxa (e.g., carrion beetles [Coleoptera: Silphidae]) by effectively acting as bait.

The possibility for organisms to escape from traps is worth mentioning at this stage as rates of escape have been shown to vary with the material from which the trap is constructed (Luff 1975), and presumably will also vary with design. For example, Petruska (1969) reported it was possible for arthropods to escape glass pitfall traps containing a solution of formalin (although he did not state how far the trap rim was from the level of killing preservative which may influence escape rates relative to body size and mobility). Additionally, Yamashita et al. (2010) reported sex‐biased differences in the rate of escape, despite similar rates of initial capture, when using dry pitfall traps in mark‐recapture studies. As capture rates may not equal retention rates, they should be considered separately. While capture rates are more difficult to optimize (they will vary with species and in relation to the other design features, as well as environmental conditions), it should be possible to optimize retention so that reported samples represent as close to 100% as possible of the organisms initially captured. It is likely that funnels reduce the possibility of escape as they present an additional overhanging barrier to organisms captured in the pitfall trap, and this could be easily quantified in simple laboratory trials.

From the 60 publications, we examined whether it was possible to determine the presence or absence of funnels in only 10% of them (Fig. 2). Of the 6 publications where the presence or absence of funnel traps could be determined, one‐third of researchers utilized a funnel pitfall trap design. While it is likely that publications not stating the use of funnel used the conventional nonfunnel design, we propose that funnels be included in future research on the basis that they reduce vertebrate bycatch and reduce fouling or differential attraction effects.

## Use of Rain Guards

Rain guards on pitfall traps have a sporadic history of use and have been made from various materials including asbestos, wood, plastic, metal, and natural materials such as leaves (Olson 1994). Generally, rain guards are intended to either reduce evaporation rate of killing preservatives or to reduce desiccation of captured organisms, as well as to reduce the pollution of traps by wind‐blown leafy debris and rainfall (e.g., Fichter 1941).

Buchholz and Hannig (2009) field tested whether different colors of rain guards (and presence of rain guards overall) would influence captures of various arthropod taxa, reporting no significant differences in capture rates for ants, beetles, or spiders, concluding that the use of rain guards posed no significant influence on trapping efficiency. However, earlier work by Joosse (1965) reported that responses to transparent and asbestos (shade casting) rain guards varied between four species of Collembolan. More recent research by Bell et al. (2014) supported the use of transparent rain guards after discovering differences in capture rates of Carabid beetles between opaque and transparent rain guards. In conjunction with the background heterogeneity hypothesis presented by Work et al. (2002), and the effects of pitfall color reported by (Buchholz et al. 2009), we agree with their recommendation and also advocate the use of transparent rain guards for a standardized pitfall trap design. While in some instances the effect of rain guard color has been shown to not significantly influence capture rates of certain taxa, it seems likely that this effect will be difficult to predict and again we suggest that it may be best to simply avoid guessing altogether and use a standard transparent rain guard.

In the surveyed literature, 33% of authors reported whether they used rain guards or not (Fig. 2). Of the 20 publications using rain guards, only one did not state what the rain guard was constructed from, although we saw considerable variation in construction material and degree of transparency. Materials varied, including use of galvanized metal (Bowie et al. 2014), plywood tiles (Guarisco et al. 2004), and plastic (Furlong et al. 2004).

The benefits of using rain guards in terms of reducing killing preservative dilution and leaf litter accumulation in traps (which will be likely to influence escape rates) would suggest that using transparent rain guards, at a fixed distance above traps, would improve comparability between studies. The rain guards used should be at least of the same diameter of the pitfall trap itself. These can be easily and cheaply constructed from large petri dishes and suspended using nails or stakes.

## Pitfall Trap Size

There are two basic components to pitfall trap size – the diameter of the trap opening and the trap depth. Each can intuitively influence both the rate of capture and retention of specimens, although to date most attention has been aimed at the diameter of pitfall traps and the relation between trap size and rate of capture. In an in effort to determine an optimal size, several authors have investigated the effects of pitfall trap diameter on captures of different taxa (Table S3).

The depth of pitfall traps has received comparatively little research attention although generally larger pitfall traps are also deeper owing to the use of plastic drinking cups as a common construction material. Pendola and New (2007) assessed the influence of depth of pitfall traps on captures of ants and reported similar species compositions when using “shallow” traps compared to “deep” traps (150 and 80 mm depth). They suggested shallow traps could be reliably utilized for rapid biodiversity monitoring where small vertebrate bycatch was an issue. There is presumably an upper and lower limit where depth either has no additional effect on escapability or greatly limits the capture potential of the pitfall trap.

In the meta‐analysis, 73% of publications we examined reported the diameter of the pitfall traps used (Fig. 3). The diameter of pitfall traps in use ranged from 18 mm to 185 mm and median of 52 mm. The number of publications not stating a trap diameter was consistent between 1994 and 2004 (6%), but was higher in 2014 (13%). Trap depth was reported comparatively less often (43%), although the rate of nonreporting was consistent at around 50% in 1994, 2004, and 2014. The depth of pitfall traps (mm) ranged from 55 mm to 200 mm, with median of 95 mm.

## Choice of Killing Preservative

The choice of a killing preservative has been a source of considerable debate in the literature, and we fully appreciate that depending on the aims of the research (e.g., morphology or genomic focus), the ability to standardize the killing preservative is probably less easily achieved than for the other design features discussed so far. Those wishing to collect material for genetic investigation will have different priorities than those solely wishing to investigate morphological features, who may get away with using cheaper killing preservatives or those more resistant to evaporation. External considerations such as the disposal regulations of killing preservatives are also relevant, as some substances historically used as pitfall trap killing preservatives are toxic or environmentally damaging should they be improperly disposed of or if the traps are prone to frequent flooding (Braun et al. 2009).

While flexibility in preservative use is to be expected owing to differences in study aims, a move toward reducing the number of killing preservatives in use is not impossible. Several studies have investigated the merits of a variety of killing preservatives, and these are summarized in Table S4. It is clear from the surveyed literature that the use of killing preservatives is almost completely nonstandardized; of 60 surveyed papers that reported using a killing preservative, there were 11 distinct categories of killing preservative in use (these categories being defined by the major additive to aqueous solution – e.g., propylene/ethylene glycols, ethanol, salts and formalin), in addition to the variation arising from differences in dilution and other additives within these categories (Fig. 2). In addition, the volume of killing preservative was nonstandardized – presumably, the depth of solution and the distance from the trap rim could influence retention of samples.

The problem in suggesting a standardized killing preservative is that both the literature on the preservation ability of different preservatives and the techniques to extract DNA from old or degraded specimens is advancing rapidly. For example, Pokluda et al. (2014) have recently shown that 2% SDS and 100 mmol/L EDTA solutions are capable of preserving the DNA of Coleoptera for up to 8 weeks and recommend these for use by entomologists interested in collecting material for barcoding, while Miller et al. (2013) described a method that allowed extraction of DNA from arachnid specimens stored in 70% ethanol for up to 50 years (with varying success depending on the age and body size of the specimen). The effort of undertaking field studies to investigate how these new preservatives influence capture rates represents a considerable challenge. While the recommendation of a single killing preservative is perhaps not possible at this stage, we suggest that some categories of killing preservatives are at the very least removed from general use. This would include formalin, sodium benzoate solutions, ethylene glycol, and “household” materials such as saturated salt solution or wine/vinegars.

From the 60 surveyed publications, 11% did not state whether a killing preservative was used or not, while 15% of the publications we examined did not use any killing preservative and focused on capturing living specimens (Fig. 2). The most common killing preservative used was ethylene glycol (18% of studies), which varied in concentration from 100% to 30% in water. Formalin of differing strengths ranging from 4% to 10% was encountered in 17% of the studies that reported using a killing preservative. Propylene glycol was found in 5 publications between 20% and 30% concentrations. We encountered 4 publications where the killing preservative varied during the research (between years).

Finally, we wish to make a small point concerning reporting clarity specific to killing preservatives, as in some cases the mix and concentration of preservatives was open to interpretation. For example, if the killing preservative used was a “70% ethanol and 30% glycerol mix”, this can be interpreted in several ways (e.g., 70% ethanol solution with a 30% v/v addition of neat glycerol or a mix of neat ethanol and neat glycerol mixed 7:3). It would be preferable to report killing preservatives in a clear manner, stating the parts first and then the concentrations of each component (e.g., “7 parts 70% ethanol [aq.] with 3 parts neat glycerol”).

## Reporting of Experimental Data of Relevance to Biodiversity Research

In the surveyed literature, we found numerous instances of missing or poorly reported data that would potentially reduce the value of these studies to future meta‐analysis or “big data” interests (Figs. 2, 3, 4).

In order to improve reporting and facilitate comparisons, we recommend the following template table (Table S5) of methodological details should be reported as standard, in the supplementary material if word limits are restrictive. If the reporting of such data were to be standardized, it would be easier to automate the mining of this data for future meta‐analysis (e.g., Lajeunesse 2015).

## A Standard Biodiversity Monitoring Pitfall Trap

The bottom line is there is no universal “best” design (van den Berghe 1992).

Thirty‐seven years after Adis (1979) first highlighted the need for a standardized pitfall trap methodology, we are still awaiting consensus (Fig. 5). However, it is hoped this review and proposal represent a first step toward a more unified, comparable methodology. Sufficient literature exists identifying a range of biases that influence the capture and retention rates of pitfall traps, suggesting that pitfall trap design needs to be standardized. The design of pitfall traps is completely within the control of entomologists and a standardized design would allow these biases to be further investigated and understood. In addition, the use of a standardized design of biodiversity pitfall trap would facilitate the optimization of sampling protocol (e.g., the spatial arrangement and intensity of trapping). Additionally, as long‐term datasets become increasingly critical for biodiversity and conservation research (Magurran et al. 2010; Dornelas et al. 2014), a standardized design of one of the most commonly used techniques used by entomologists will allow easier generation of large‐scale, long‐term datasets. Such data are unlikely to be generated by individuals, and therefore broad comparability and repeatability are of vital importance. We expect a similar rationale could be applied to other ecological methods, facilitating future macro‐analysis.

While we recognize that in some instances the needs of individual research projects will dictate variation in design, we propose the use of a standardized design as an opt‐in method that will add value to research where the use of the design does not compromise the main research goals. Our standard pitfall trap design is necessarily tailored toward sampling the taxonomic groups most commonly sampled by pitfall traps; Coleoptera, Araneae, and Formicidae. While other organisms (e.g., Collembola, Diptera, Diplopoda) are collected by pitfall traps, it is debateable that pitfall trapping would represent the optimal method for their collection, especially when more efficient collection methods exist and seem to be more widely used (e.g., litter sieving, Winkler bags and Tullgren extraction,).

In general, a transparent plastic pitfall trap, of ca. 11 cm diameter, with an inner sampling pot and using a nontoxic killing preservative seems well supported for general use collecting Araneae, Coleoptera, and Formicidae. Our research experience is admittedly catering to the European fauna, but aside from perhaps a requirement for larger diameter and deeper pitfall traps to deal with larger body‐size arthropod species found the tropics, the same biases and rationale for this design should apply globally. We recognize that in some cases this pitfall design will not represent the optimal design – for example, when sampling tidal mud flats (Mertens et al. 2007) or under snow (Steigen 1973). But these situations are quite specialized, and there is no reason not to standardize within those applications such that when “snow pitfall traps” are used, they are not unique to each different research group. Some standardization is likely better than none.

Following the rationale above, it is proposed that a “standardized pitfall trap for biodiversity monitoring” for the generation of long‐term and spatially large ecological datasets should implement the following design features (Table 1). The rationale for using a two‐cup design is based on ease of installation of the pitfall trap so that the rim is level with the soil surface. Using two identical outer containers nested in one another during trap installation allows the soil and other debris to be more easily removed prior to commencing collection of specimens. A further improvement to this can be made using a third container, with a screw top lid, during sampling. This container should contain the killing preservative and fit within the outer cup and beneath the funnel (Fig. S1). Using screw top sample containers avoids the need to decant samples in the field and significantly speeds up collection of samples.

**Table 1:** Design features of the proposed standard pitfall trap

Material	Diameter	Depth	Color	Use of funnel	Rain guard	Killing preservative
Plastic, 2‐cup design	90–110 mm	90–110 mm	Transparent	Transparent. Report funnel opening diameter.	Transparent. Report diameter and height above trap.	100 ml of a suitable transparent, nontoxic killing preservative such as propylene glycol, with concentration clearly reported.

## Conflict of Interest

The authors declare they have no conflict of interests.

## Supporting information


**Figure S1.** (a) Schematic drawing of the standardised pitfall trap proposed in this review showing the assembly in exploded and operational views. (b) Photograph of the proposed standardised pitfall trap. (c) Photograph of the trap components.

**Table S1.** List of papers used in the meta‐analysis.

**Table S2.** Data used in the meta‐analysis.

**Table S3.** A survey of pitfall trap diameters investigated by previous researchers, with their suggested optimal diameter (**Ø**, mm) reproduced.

**Table S4.** A survey of killing preservatives previously investigated for use in arthropod pitfall trapping research, with each author's suggested optimal killing preservative highlighted.

**Table S5.** Pitfall methodology reporting template.
