# Supplementary material for: A review of extensive variation in the design of pitfall traps and a proposal for a standard pitfall trap design for monitoring ground‐active arthropod biodiversity
Source: Ecol Evol. 2016 May 12;6(12):3953–64. doi: 10.1002/ece3.2176 (PMC4867678; doi:10.1002/ece3.2176)
Supplement: Supplementary file 1 — Figure S1. (a) Schematic drawing of the standardised pitfall trap proposed in this review showing the assembly in exploded and operational views. (b) Photograph of the proposed standardised pitfall trap. (c) Photograph of the trap components. [file ECE3-6-3953-s001.docx]

Supplementary figure 1


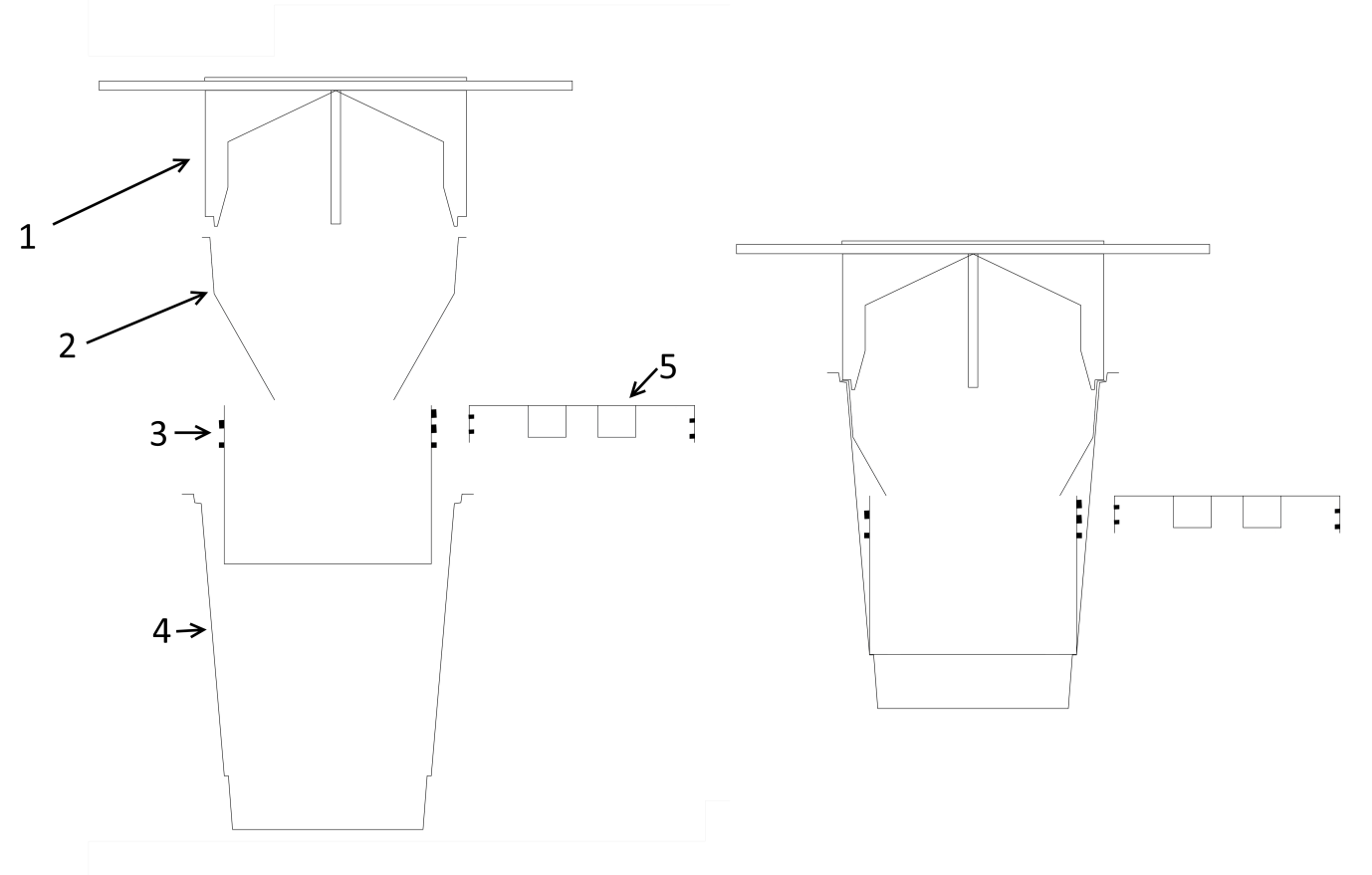


Figure 1a. Schematic drawing of the standardised pitfall trap proposed in this review showing the assembly in exploded and operational views. 1 = Rain guard (a large petri dish or similar), 2 = funnel, 3 = inner sample collection pot (screw threaded), 4 = Outer collection pot, 5 = screw top lid for (3). By exchanging pre-labelled screw top inner sample containers with preservative solution already measured into them, collecting the samples becomes more efficient as there is no need to decant preservatives and specimens in the field. This also eliminates the problem of specimens adhering to the collection container or being lost during transfer to sample pots.

**List of suppliers**

We would like to state we have no affiliation with any of the suppliers listed below, and they are noted simply for convenience (and these are the suppliers we have used to source our trap components in previous research). We give indication of price for each component (approximate at the time of manuscript submission, January 2016).

**Outer collection cup:** SOLO® Brand SMOOTHIE CUPS 12-14oz.

Available from several suppliers in the UK and elsewhere. Typical pack size is 1000 cups (£50-60).

**Inner collection sample pot:** Wide Neck Jar, polypropylene. Dimensions: 150ml capacity Diameter 65mm, Height 50mm. Available from [www.dormex.co.uk](http://www.dormex.co.uk) – product code: D8011N

Lids to fit are available from the same supplier (product code C1183W). We typically purchased multiples of 100 pots (ca. £15/100).

**Funnel**: 75mm plastic funnels from Stewart Plastics will fit exactly into the inner rim of the solo plastic smoothie cups. We bought ours directly from Stewart Plastics (www. stewartcompany.co.uk). Cost when purchased (2011) was 47p per funnel. They require trimming to remove the lower portion of the funnel.

**Rain-guard**: At present we know of no purpose made rain-guard. We suggest using large petri dishes as rain-guards, which can be held above the trap using wooden skewers. Petri dishes of appropriate size (150mm diameter) can be sourced from a variety of laboratory suppliers (Sigma, Fisher, Carl Roth, etc.). In the photographs below the rain-guard has been constructed from thin acrylic plastic.


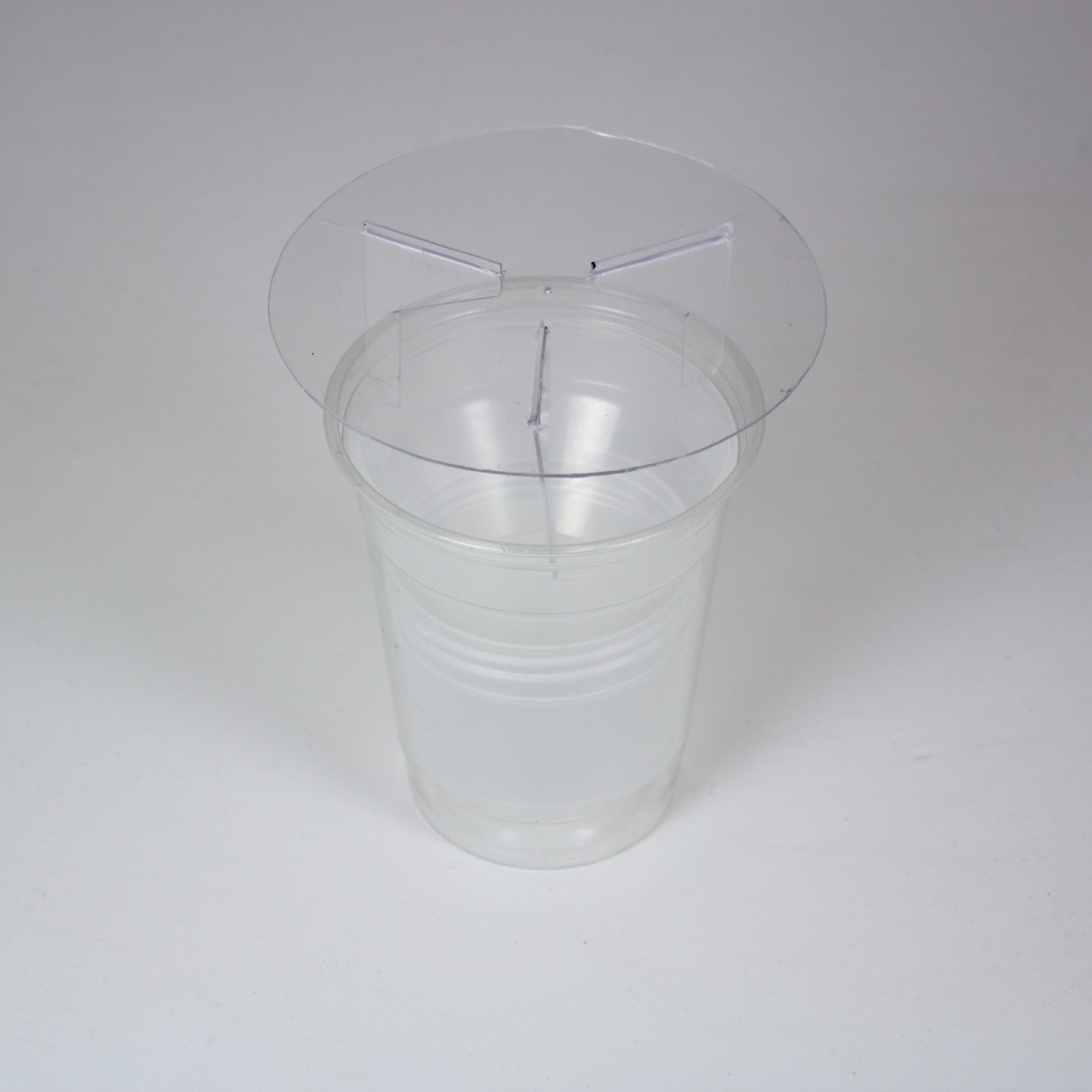
Figure 1b. Photograph of the proposed standardised pitfall trap. The rain-guard is 130mm diameter and held 40mm above the trap. The advantage of a rain-guard with attached legs is that it avoids the need to measure and level rain-guards during field installation and sample collection.
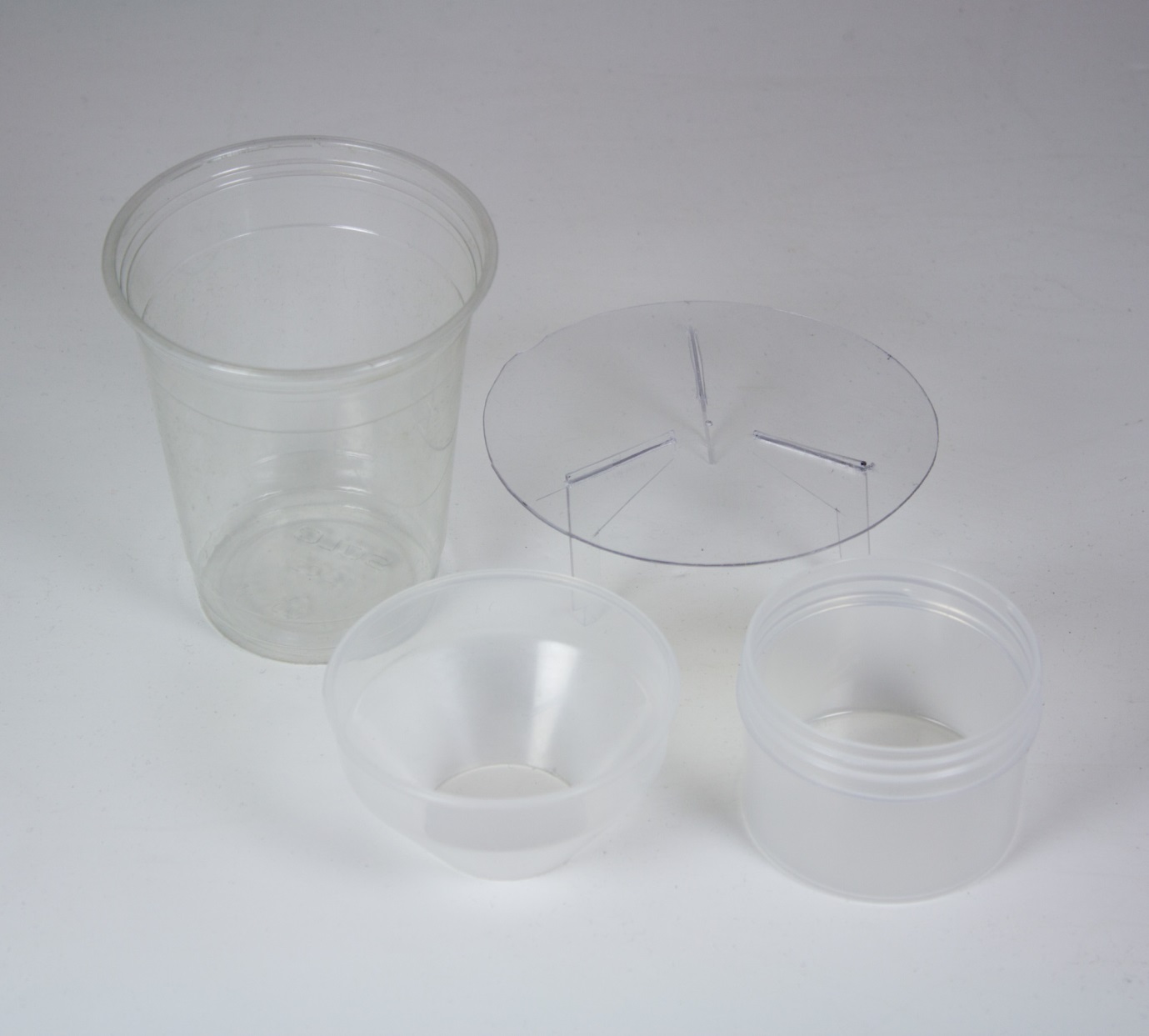
Figure 1c. Photograph of the trap components (clockwise from left: Outer pot, rain-guard, inner sample pot with screw threads, funnel).
