# Supplementary material for: A review of extensive variation in the design of pitfall traps and a proposal for a standard pitfall trap design for monitoring ground‐active arthropod biodiversity
Source: Ecol Evol. 2016 May 12;6(12):3953–64. doi: 10.1002/ece3.2176 (PMC4867678; doi:10.1002/ece3.2176)
Supplement: Supplementary file 2 — Table S1. List of papers used in the meta‐analysis. [file ECE3-6-3953-s002.docx]

**Supplementary Table 1**

List of papers used in the meta-analysis

Anderson, S.J., Emberson, R.M. & Brown, B. (2004) Seasonal activity and habitat associations of Mecodema howitti and Megadromus guerinii, two endemic New Zealand ground beetles (Coleoptera: Carabidae). New Zealand Journal of Zoology, 31, 305-312.

Baker, S.C., Richardson, A.M.M., Seeman, O.D. & Barmuta, L.A. (2004) Does clearfell, burn and sow silviculture mimic the effect of wildfire? A field study and review using litter beetles. Forest Ecology and Management, 199, 433-448.

Barragán, F., Moreno, C.E., Escobar, F., Bueno-Villegas, J. & Halffter, G. (2014) The impact of grazing on dung beetle diversity depends on both biogeographical and ecological context. Journal of Biogeography, 41, 1991-2002.

Batzer, D. (2004) Movements of upland invertebrates into drying seasonal woodland ponds in northern Minnesota, USA. Wetlands, 24, 904-907.

Bowie, M.H., Klimaszewski, J., Vink, C.J., Hodge, S. & Wratten, S.D. (2014) Effect of boundary type and season on predatory arthropods associated with field margins on New Zealand farmland. New Zealand Journal of Zoology, 41, 268-284.

Bugs, P.d.S., Araujo, P.B., Mendonça Júnior, M.d.S. & Ott, R. (2014) Diversity and population characteristics of terrestrial isopods (Crustacea, Oniscidea) across three forest environments in southern Brazil. Iheringia. Série Zoologia, 104, 334-340.

Cardarelli, E. & Bogliani, G. (2014) Effects of grass management intensity on ground beetle assemblages in rice field banks. Agriculture, Ecosystems & Environment, 195, 120-126.

Cederbaum, S.B., Carroll, J.P. & Cooper, R.J. (2004) Effects of Alternative Cotton Agriculture on Avian and Arthropod Populations. Conservation Biology, 18, 1272-1282.

Da Costa, C.M.Q., Barretto, J.W. & de Moura, R.d. (2014) Changes in the dung beetle community in response to restinga forest degradation. Journal of Insect Conservation, 18, 895-902.

Dave, D., Hamer, M., Slatow, R. & Prendini, L. (2004) Checklist of millipedes (Diplopoda), centipedes (Chilopoda) and scorpions (Arachnida: Scorpionida) from a savanna ecosystem, Limpopo Province, South Africa. African Invertebrates, 45, 315-322.

Frank, S.D. & Shrewsbury, P.M. (2004) Effect of Conservation Strips on the Abundance and Distribution of Natural Enemies and Predation of Agrotis ipsilon (Lepidoptera: Noctuidae) on Golf Course Fairways. Environmental Entomology, 33, 1662-1672.

Furlong, M.J., Zu-Hua, S., Yin-Quan, L., Shi-Jian, G., Yao-Bin, L., Shu-Sheng, L. & Zalucki, M.P. (2004) Experimental Analysis of the Influence of Pest Management Practice on the Efficacy of an Endemic Arthropod Natural Enemy Complex of the Diamondback Moth. Journal of Economic Entomology, 97, 1814-1827.

Gómez, A.d.l.S., Nicolás, J.P.d. & Dorta-Guerra, R. (2014) Abundance, biomass and diversity of ground-beetles (Col. Carabidae) as indicators of climatic change effects over elevation strata in Tenerife (Canary Islands). Ecological Indicators, 46, 504-513.

Guarisco, H., Cook, W.M. & Nuckolls, K.R. (2004) New Additions to the Spider Fauna of Kansas Discovered near Black-Tailed Prairie Dog Towns in Shortgrass Prairie. Transactions of the Kansas Academy of Science (1903-), 107, 175-178.

Gunnarsson, B., Nittérus, K. & Wirdenäs, P. (2004) Effects of logging residue removal on ground-active beetles in temperate forests. Forest Ecology and Management, 201, 229-239.

HORVÁTH, R., ELEK, Z. & LÖVEI, G.L. (2014) Compositional changes in spider (Araneae) assemblages along an urbanisation gradient near a Danish town. Bulletin of Insectology, 67, 255-264.

Ipser, R.M., Brinkman, M.A., Gardner, W.A. & Peeler, H.B. (2004) A Survey of Ground-Dwelling Ants (Hymenoptera: Formicidae) in Georgia. Florida Entomologist, 87, 253-260.

Johnson, J.M., Hough-Goldstein, J.A. & Vangessel, M.J. (2004) Effects of Straw Mulch on Pest Insects, Predators, and Weeds in Watermelons and Potatoes. Environmental Entomology, 33, 1632-1643.

Kiss, O., Elek, Z. & Moskát, C. (2014) High breeding performance of European Rollers Coracias garrulus in heterogeneous farmland habitat in southern Hungary. Bird Study, 61, 496-505.

Koivula, M., Hyyryläinen, V. & Soininen, E. (2004) Carabid beetles (Coleoptera: Carabidae) at forest-farmland edges in southern Finland. Journal of Insect Conservation, 8, 297-309.

Kyerematen, R., Owusu, E.H., Acquah-Lamptey, D., Anderson, R.S. & Ntiamoa-Baidu, Y. (2014) Species Composition and Diversity of Insects of the Kogyae Strict Nature Reserve in Ghana. Open Journal of Ecology, 4, 1061.

Langhans, S.D. & Tockner, K. (2014) Edge Effects Are Important in Supporting Beetle Biodiversity in a Gravel-Bed River Floodplain. Plos One, 9, e114415.

Lemieux, J.P. & Lindgren, B.S. (2004) Ground beetle responses to patch retention harvesting in high elevation forests of British Columbia. Ecography, 27, 557-566.

Leroy, B., Le Viol, I. & Pétillon, J. (2014) Complementarity of rarity, specialisation and functional diversity metrics to assess community responses to environmental changes, using an example of spider communities in salt marshes. Ecological Indicators, 46, 351-357.

Milberg, P., Bergman, K.-O., Johansson, H. & Jansson, N. (2014) Low host-tree preferences among saproxylic beetles: a comparison of four deciduous species. Insect Conservation and Diversity, 7, 508-522.

Morrison, L.W. (2004) Spatiotemporal Variation in Antlion (Neuroptera: Myrmeleontidae) Density and Impacts on Ant (Hymenoptera: Formicidae) and Generalized Arthropod Foraging. Annals of the Entomological Society of America, 97, 913-922.

Nash, M.S., Bradford, D.F., Franson, S.E., Neale, A.C., Whitford, W.G. & Heggem, D.T. (2004) Livestock grazing effects on ant communities in the eastern Mojave Desert, USA. Ecological Indicators, 4, 199-213.

Price, D.L. (2004) Species diversity and seasonal abundance of scarabaeoid dung beetles (coleoptera: scarabaeidae, geotrupidae and trogidae) attracted to cow dung in central new jersey. Journal of the New York Entomological Society, 112, 334-347.

Quevedo, L., Arnan, X., Boet, O. & Rodrigo, A. (2014) Post-fire selective thinning of Arbutus unedo L. Coppices keeps animal diversity unchanged: the case of ants. Annals of Forest Science, 71, 897-905.

Samu, F. & Urák, I. (2014) Are more bogs better? Comparative studies into Transylvanian peat bog spider (Arachnida: Araneae) assemblages from a conservation biological perspective. North-Western Journal of Zoology, 10, S94-S101.

Sas-Kovacs, E.-H. & Sas-Kovacs, I. (2014) Lycosidae (Arachnida: Araneae) in “Câmpia Careiului”(north-western Romania): preliminary assessment of composition, distribution, habitat preference and conservation. North-Western Journal of Zoology, 10, S102-S114.

Sinclair, J.E. & New, T.R. (2004) Pine plantations in south eastern Australia support highly impoverished ant assemblages (Hymenoptera: Formicidae). Journal of Insect Conservation, 8, 277-286.

Staab, M., Schuldt, A., Assmann, T., Bruelheide, H. & Klein, A.-M. (2014) Ant community structure during forest succession in a subtropical forest in South-East China. Acta Oecologica, 61, 32-40.

TABOADA, A., KOTZE, D.J. & SALGADO, J.M. (2004) Carabid beetle occurrence at the edges of oak and beech forests in NW Spain. European Journal of Entomology, 101, 555-563.

Urbanovičová, V., Miklisová, D., Mock, A. & Kováč, L.u.r. (2014) Activity of epigeic arthropods in differently managed windthrown forest stands in the High Tatra Mts. North-Western Journal of Zoology, 10, 337–345.

Van Hamburg, H., Andersen, A.N., Meyer, W.J. & Robertson, H.G. (2004) Ant Community Development on Rehabilitated Ash Dams in the South African Highveld. Restoration Ecology, 12, 552-558.

Vasconcelos, H.L., Frizzo, T.L.M., Pacheco, R., Maravalhas, J.B., Camacho, G.P., Carvalho, K.S., Koch, E.B.A. & Pujol-Luz, J.R. (2014) Evaluating sampling sufficiency and the use of surrogates for assessing ant diversity in a Neotropical biodiversity hotspot. Ecological Indicators, 46, 286-292.

Wiebe, A.P. & Obrycki, J.J. (2004) Quantitative assessment of predation of eggs and larvae of Galerucella pusilla in Iowa. Biological Control, 31, 16-28.

Zauli, A., Chiari, S., Hedenström, E., Svensson, G. & Carpaneto, G. (2014) Using odour traps for population monitoring and dispersal analysis of the threatened saproxylic beetles Osmoderma eremita and Elater ferrugineus in central Italy. Journal of Insect Conservation, 18, 801-813.

Zhang, B., Chang, L., Ni, Z., Callaham Jr, M.A., Sun, X. & Wu, D. (2014) Effects of land use changes on winter-active Collembola in Sanjiang Plain of China. Applied Soil Ecology, 83, 51-58.
