# Supplementary material for: A review of extensive variation in the design of pitfall traps and a proposal for a standard pitfall trap design for monitoring ground‐active arthropod biodiversity
Source: Ecol Evol. 2016 May 12;6(12):3953–64. doi: 10.1002/ece3.2176 (PMC4867678; doi:10.1002/ece3.2176)
Supplement: Supplementary file 4 — Table S3. A survey of pitfall trap diameters investigated by previous researchers, with their suggested optimal diameter (Ø, mm) reproduced. [file ECE3-6-3953-s004.docx]

**Supplementary Table 3**

A survey of pitfall trap diameters investigated by previous researchers, with their suggested optimal diameter (**Ø**, mm) reproduced.

| **Taxon** | **Author** | **Trap materials used** | **Killing preservative used** | **Trap Ø investigated (mm)** | **‘Optimal’ trap Ø (mm)** |
| --- | --- | --- | --- | --- | --- |
| Formicidae | Abensperg-Traun & Steven, 1995 | Glass, plastic | Propylene glycol | 18, 42, 86, 135 | 86, 135 |
| Carabidae, Tenebrionidae | Morrill et al., 1990 | Plastic | None | 173, 251; 140,160,180,200,220 | 251, 220 |
| Formicidae | Borgelt & New, 2005 | Glass, plastic | 70% Ethanol | 18, 70 | 70 |
| Araneae | Brennen et al., 2005 | Plastic | Gault's solution and 2ml detergent | 43, 70, 111 | 111 |
| Carabidae | Benest, 1989 | Glass, Aluminium | None | 50,80, "600mm L-shaped" | 600 |
| Carabidae | Luff, 1975 | Glass, plastic | None | 25, 50, 65, 100 | 65 |
| Carabidae, Staphylinidae, Araneae | Work et al., 2002 | Plastic | Ethylene glycol | 45, 65, 110, 150, 200 | >110 |
| Carabidae | Koivula et al., 2003 | Plastic | Vinegar, Propylene glycol | 65, 90 | 90 |

**Table references**

Abensperg-Traun, M. & Steven, D. (1995) The Effects Of Pitfall Trap Diameter On Ant Species Richness (Hymenoptera: Formicidae) And Species Composition Of The Catch In A Semi-Arid Eucalypt Woodland. Australian Journal Of Ecology, 20, 282-282.

Benest, G. (1989) The Sampling Of A Carabid Community: I. The Behaviour Of A Carabid When Facing The Trap. Revue D'écologie Et De Biologie Du Sol, 26, 205-211.

Borgelt, A. & New, T., R (2005) Pitfall Trapping For Ants (Hymenoptera, Formicidae) In Mesic Australia: The Influence Of Trap Diameter. Journal Of Insect Conservation, 9, 219-221.

Brennan, K.E.C., Majer, J.D. & Moir, M.L. (2005) Refining Sampling Protocols For Inventorying Invertebrate Biodiversity: Influence Of Drift-Fence Length And Pitfall Trap Diameter On Spiders. Journal Of Arachnology, 33, 681-702.

Koivula, M., Kotze, D.J., Hiisivuori, L. & Rita, H. (2003) Pitfall Trap Efficiency: Do Trap Size, Collecting Fluid And Vegetation Structure Matter? Entomologica Fennica, 14, 1-14.

Luff, M.L. (1975) Some Features Influencing The Efficiency Of Pitfall Traps. Oecologia, 19, 345-357.

Morrill, W., Lester, D. & Wrona, A. (1990) Factors Affecting Efficacy Of Pitfall Traps For Beetles (Coleoptera: Carabidae And Tenebrionidae). Journal Of Entomological Science, 25, 284-293.

Work, T.T., Buddle, C.M., Korinus, L.M. & Spence, J.R. (2002) Pitfall Trap Size And Capture Of Three Taxa Of Litter-Dwelling Arthropods: Implications For Biodiversity Studies. Environmental Entomology, 31, 438-448.
