# Supplementary material for: A review of extensive variation in the design of pitfall traps and a proposal for a standard pitfall trap design for monitoring ground‐active arthropod biodiversity
Source: Ecol Evol. 2016 May 12;6(12):3953–64. doi: 10.1002/ece3.2176 (PMC4867678; doi:10.1002/ece3.2176)
Supplement: Supplementary file 5 — Table S4. A survey of killing preservatives previously investigated for use in arthropod pitfall trapping research, with each author's suggested optimal killing preservative highlighted. [file ECE3-6-3953-s005.docx]

**Supplementary Table 4**

A survey of killing preservatives previously investigated for use in arthropod pitfall trapping research, with each author's suggested optimal killing preservative highlighted.

| Taxa | Author | Killing preservative investigated | Purpose | ‘Optimal’ |
| --- | --- | --- | --- | --- |
| Scarabaeoidea | Aristophanous, 2012 | Brine, saturated borax solution, propylene glycol (33%, 50%, 75%), white vinegar, 100% ethylene glycol, FAACC, 4% buffered formaldehyde, 96% ethanol | Comparison of killing preservative for preserving dung beetle reproductive organs | 4% formaldehyde |
| Araneae, Carabidae | Schmidt et al., 2006 | Water, brine, 67% ethanol, 75% ethanol and 25% glycerin, 33% ethylene glycol | Testing efficiency of capture and preservation of different killing preservatives | 33% ethylene glycol |
| Araneae | Jud and Schmidt-Entling, 2008 | 100% ethylene glycol, 25% ethylene glycol, 100% propylene glycol, 25% propylene glycol, 2% formalin (all killing preservatives +/- bittering agent) | Comparison of killing preservative efficacy and bitter agent effect | 2% formalin / diluted glycols |
| Araneae, Coleoptera | Weeks and McIntyre, 1997 | (none), water, 100% ethylene glycol, 100% propylene glycol | Comparison of killing preservative and non- lethal trapping | Propylene glycol |
| Araneae | Topping and Luff 1995 | Ethylene glycol (100%, 1/24 dilution with water), water +/- detergent | Effects of detergent and dilution on capture efficacy of spiders | Dilution not significant but addition of detergent deemed necessary |
| Carabidae | Koivula et al., 2003 | 75% ethylene glycol, commercial antifreeze, commercial paraffin, salt water | Capture efficiency of different killing preservatives in pitfall traps | 75% ethylene glycol |
| Arachnids, Coleoptera | Pekar, 2002 | 1,2,4,10,20% formalin +/- detergent | Effects of formalin concentration of capture efficacy of pitfall traps | Varied by taxa, but >4% formalin without detergent suggested as optimal |
| Hemiptera: Pyrrhocoridae | Braun et al., 2009 | Ethylene glycol (various grades) | Assessment of various grades of ethylene glycol on insect sample preservation | Technical grade ethylene |
| Araneae, Opiliones | Curtis, 1980 | Dry, Detergent only, 4% formalin + detergent | Comparison of capture rates of pitfall killing preservatives vs. dry | 4% formalin plus detergent |
| Araneae | Gurdebeke and Maelfait, 2002 | 70% ethanol, 1:1 acetic acid : TE buffer, 4% formaldehyde | Efficacy of different solutions for subsequent DNA extraction from pitfall samples | 75% ethanol |
| Carabidae | Vennila and Rajagopal, 2000 | 4% formalin, 2% ethylene glycol, 20% salt solution, 2% detergent solution | Comparison of trap material, sample frequency and killing preservative used on captures | 4% formalin or 2% ethylene glycol |
| Araneae | Vink et al. 2005 | RNAlater, propylene glycol, ethanol (various concentrations) | Effect of temperatures and concentration of killing preservative on arachnid DNA | RNAlater or propylene glycol |
| Formicidae | Greenslade and Greenslade, 1971 | Commercial methylated spirit, ethanol-glycerol mix, water | Comparison of various killing preservatives, baits and use of dry traps in sampling ant populations (+ influence on other taxa) | Methylated spirit |
| Carabidae | Sasakawa, 2007 | Brine, 10% acetic acid, 100% ethylene glycol | Effect of killing preservative on specimen condition for taxonomic investigation | None; Acetic acid |

**Table references**

Aristophanous, M. (2010) Does Your Preservative Preserve? A Comparison Of The Efficacy Of Some Pitfall Trap Solutions In Preserving The Internal Reproductive Organs Of Dung Beetles. Zookeys, 34, 1-16.

Braun, M., Simon, E., Fabian, I. & Tothmeresz, B. (2009) The Effects Of Ethylene Glycol And Ethanol On The Body Mass And Elemental Composition Of Insects Collected With Pitfall Traps. Chemosphere, 77, 1447-1452.

Braun, M., Simon, E., Fabian, I. & Tothmeresz, B. (2012) Elemental Analysis Of Pitfall-Trapped Insect Samples: Effects Of Ethylene Glycol Grades. Entomologia Experimentalis Et Applicata, 143, 89-94.

Curtis, D.J. (1980) Pitfalls In Spider Community Studies (Arachnida, Araneae). Journal Of Arachnology, 8, 271-280.

Greenslade, P. & Greenslade, P.J.M. (1971) The Use Of Baits And Preservatives In Pitfall Traps. Australian Journal Of Entomology, 10, 253-260.

Greenslade, P. & Greensladed, P.J.M. (1971) The Use Of Baits And Preservatives In Pitfall Traps. Australian Journal Of Entomology, 10, 253-260.

Gurdebeke, S. & Maelfait, J.-P. (2002) Pitfall Trapping In Population Genetics Studies: Finding The Right "Solution". Journal Of Arachnology, 30, 255-261.

Jud, P. & Schmidt-Entling, M.H. (2008) Fluid Type, Dilution, And Bitter Agent Influence Spider Preservation In Pitfall Traps. Entomologia Experimentalis Et Applicata, 129, 356-359.

Koivula, M., Kotze, D.J., Hiisivuori, L. & Rita, H. (2003) Pitfall Trap Efficiency: Do Trap Size, Collecting Fluid And Vegetation Structure Matter? Entomologica Fennica, 14, 1-14.

Pekár, S. (2002) Differential Effects Of Formaldehyde Concentration And Detergent On The Catching Efficiency Of Surface Active Arthropods By Pitfall Traps. Pedobiologia, 46, 539-547.

Sasakawa, K. (2007) Effects Of Pitfall Trap Preservatives On Specimen Condition In Carabid Beetles. Entomologia Experimentalis Et Applicata, 125, 321-324.

Schmidt, M.H., Clough, Y., Schulz, W., Westphalen, A. & Tscharntke, T. (2006) Capture Efficiency And Preservation Attributes Of Different Fluids In Pitfall Traps. Journal Of Arachnology, 34, 159-162.

Topping, C.J. & Sunderland, K.D. (1992) Limitations To The Use Of Pitfall Traps In Ecological Studies Exemplified By A Study Of Spiders In A Field Of Winter Wheat. Journal Of Applied Ecology, 29, 485-491.

Vennila, S. & Rajagopal, D. (2000) Pitfall Trap Sampling Of Tropical Carabids (Carabidae: Coleoptera) - Evaluation Of Traps, Preservatives And Sampling Frequency. Journal - Bombay Natural History Society, 97, 241-246.

Vink, C.J., Thomas, S.M., Paquin, P., Hayashi, C.Y. & Hedin, M. (2005) The Effects Of Preservatives And Temperatures On Arachnid Dna. Invertebrate Systematics, 19, 99-104.

Weeks, R.D. & Mcintyre, N.E. (1997) A Comparison Of Live Versus Kill Pitfall Trapping Techniques Using Various Killing Agents. Entomologia Experimentalis Et Applicata, 82, 267-273.
