# Supplementary material for: A review of extensive variation in the design of pitfall traps and a proposal for a standard pitfall trap design for monitoring ground‐active arthropod biodiversity
Source: Ecol Evol. 2016 May 12;6(12):3953–64. doi: 10.1002/ece3.2176 (PMC4867678; doi:10.1002/ece3.2176)
Supplement: Supplementary file 6 — Table S5. Pitfall methodology reporting template. [file ECE3-6-3953-s006.docx]

**Supplementary Table 5**

Pitfall methodology reporting template

| Date(s) traps were installed |  |
| --- | --- |
| Date(s) that traps were operational |  |
| Duration of each sample (in hours) |  |
| Total number of traps in use |  |
| Total number of samples actually collected |  |
| The number of trap nights on which the analysis is based |  |
| The minimum inter-trap spacing (m) |  |
| The diameter of pitfall traps (at the opening) |  |
| The depth of the pitfall trap sample container (mm) |  |
| The colour of the pitfall trap components |  |
| The use of a rain guard |  |
| Height above the trap rain guard was installed (mm) |  |
| The volume of preservative used (ml) |  |
| The concentration and type of preservative |  |
| The use of a funnel trap design |  |
| The use of a one or two cup pitfall trap design |  |
